# Effect of smartphone use before bedtime on smartphone addiction behaviors among Chinese college students

**DOI:** 10.3389/fpsyg.2022.1023245

**Published:** 2022-10-28

**Authors:** Linghui Li, Lei Wang, Xinghua Wang

**Affiliations:** ^1^School of Mathematics and Statistics, Qingdao University, Qingdao, China; ^2^Normal College, Qingdao University, Qingdao, China

**Keywords:** problematic smartphone use, college students, sleep, smartphone addiction behavior, artificial neural network

## Abstract

Smartphone addiction behaviors are becoming more and more common with the rapid popularity and widespread use of smartphones. Such behaviors are significantly influenced by the overuse of smartphones before bedtime. In this study, the overuse of smartphones after 9:00 pm before bedtime was investigated by an online questionnaire. The sample consists of 1,035 college students in China. The artificial neural networks were applied to predict the use time of smartphones before bedtime based on their different usages, and the relationship between smartphone usage and additive behaviors was analyzed. The results show that the neural network model can more accurately predict the usage time of smartphones according to the usage of smartphones before going to bed. At the same time, it is also found that the prediction accuracy of the samples that use the smartphone for less than half an hour and more than 3 h before bed is higher than that of other samples. Moreover, watching short videos and playing games are more likely to lead to mobile phone addiction behavior. These findings would help guide college students to correctly use phones and reduce smartphone addition, which is of great significance for mental health education.

## Introduction

With the development of the Internet, the smartphone has become the largest network user terminal and its user number is also increasing. As of June 2021, the number of smartphone users has reached 3.95 billion, which accounts for 50% of the total population (Wah et al., 2022). The smartphone has brought a great impact on every aspect of people’s life (Parasuraman et al., 2017). Among them, young people are the major users of smartphones (Zhang and Wu, 2020). They have a higher ability to learn new technologies than other age groups. In China, the ages between 18 and 22 are one of the fastest-growing age groups, of which college students account for a very large proportion (Kim and Koh, 2018). College students are high-risk people for smartphone addiction because they have more free time and less supervision at college (Yu et al., 2021).

Smartphone addiction represents the physical or psychological damage to people due to the overuse of smartphones, which is also referred to as smartphone use disorder (Kardefelt-Winther, 2014). It involves symptoms observed in substance use disorders, such as withdrawal, tolerance, and reckless drug use (Billieux et al., 2015; De-Sola Gutierrez et al., 2016). Thus, smartphone addiction has some of the same clinical symptoms as other behavioral addictions, such as gambling disorder and gaming disorder (Elhai et al., 2017). Up to now, although smartphone addiction is not recognized as a type of addiction disorder in medical research community, it has caused significant damage to people’s mental health, which should be given enough attention (Elhai et al., 2020). Existing studies show that the smartphone addiction rate has reached 37.9% among 4,000 Chinese college students (Wang and Zhang, 2015).

Previous studies have shown that smartphone addiction have a harmful impact on the physical and mental health of human being. It can reduce sleep quality, impair cognitive function, and lead to loneliness (Wang et al., 2019). At the same time, it is negatively correlated with academic performance and interpersonal relationships and positively correlated with typical procrastination (Liu et al., 2018; Rozgonjuk et al., 2018; Wang et al., 2019). A 1year longitudinal study further demonstrated that frequent smartphone use among young adults increases the potential risk of sleep disturbance (Thomée et al., 2011). A series of cross-sectional studies have shown that sleep quality deteriorates with the increasing extent of smartphone addiction (e.g., Li et al., 2016). Longitudinal studies have also shown that high smartphone use is a risk factor for mental health among young adults followed by 1 year for depression and sleep disturbance (Thomée et al., 2011). There are potential mediating and regulating mechanisms in smartphone addiction and sleep quality. The effect of smartphone addiction on sleep quality and the rumination mediating effect were both moderated by mindfulness, and both effects were stronger in individuals with lower levels of mindfulness (Liu et al., 2017). Furthermore, smartphone backlighting has a disruptive effect on circadian rhythms, leading to negative sleep consequences, such as going to bed later than scheduled, thereby reducing overall sleep time (Cain et al., 2011).

However, previous studies have focused on the impact of smartphones on individual physical and mental health, as well as the direct link and potential mediating mechanisms between smartphone addiction and sleep quality. In other words, most studies have focused on the consequences associated with smartphone addiction, with a lack of exploration of factors associated with smartphone addiction. Therefore, this study conducted an in-depth exploration of the impact of smartphone use before bedtime on smartphone addiction (after 9: 00 pm). Here 9:00 pm is considered since Chinese college students usually finish their evening classes or self-study before 9:00 pm. It proposes a neural network predictive model to investigate how different smartphone usage patterns before bedtime trigger smartphone addictive behaviors. These findings could help guide college students on how to use smartphones properly and protect them from smartphone addiction.

## Materials and methods

### Participants

In order to ensure the diversity of data, corresponding questionnaire surveys were conducted on college students from five schools in the northeastern region, western region, central region and southern region of China. At the same time, the questionnaire is not randomly filled out for the students in the school, but distributed and filled out according to the grade, with the help of WeChat groups of different grades, which not only ensures the diversity of the questionnaire, but also improves the collection efficiency and quality of the questionnaire. In order to ensure the accuracy of the sample, an information prompt will be given when filling in the questionnaire. If the verification is a student, the questionnaire can continue to be filled out. In order to ensure the effectiveness of the data, 1 participant can only fill in the questionnaire once, and the same IP cannot answer repeatedly.

This study recruited participants by the online questionnaires with college students randomly selected during the academic year 2020–2021. The student participants are from provincial and subordinate colleges or universities in China; undergraduate students accounted for about 70% and graduate students accounted for about 30%. There are 1,200 students in the initial recruited sample. After removing the uncompleted data, the sample is composed of 1,035 college students. In the final sample, the participants’ mean age is equal to 22.83 years old with the range of 18 to 27 years old. The female takes 65.4% while the male takes 34.6%. After getting their permission, all of the participants were required to complete an anonymous online questionnaire.

### Measures

#### Smartphone addiction

The tested time range for using smartphones is from 9:00 pm until bedtime. We divide the duration of the use of smartphone into five groups: (1) less than half an hour, (2) half an hour to 1h, (3) 1 to 2h, (4) 2 to 3h, and (5) more than 3h. The following usages of smartphone are considered: (1) social chat, (2) watch short video, (3) play games, (4) watch TV program, (5) listen to music, (6) watch news, (7) study, (8) watch live broadcasts. (9) shop online, and (10) any others. Note that short video refers to the video playing on various new media platforms, ranging from a few seconds to several minutes. We ask participants to choose the three most commonly used ways before bedtime. The smartphone addiction scale method is used here to measure the extent of smartphone addiction. It consists of six levels, and the alpha reliability coefficient was 0.72 in the study (Zifu et al., 2021). Previous researches have demonstrated that the smartphone addiction scale method has good reliability in Chinese and is widely adopted to assess compulsive use, withdrawal, tolerance, and functional impairment (Lin et al., 2016, 2017). This scaling method consists of 10 items, such as “use smartphone longer than I expected” and “feel terrible when not using smartphone.” Based on these 10 items, we can calculate the total score and then assess the smartphone addiction behavior. The higher the total score, the higher the smartphone addiction tendency.

#### Measurement of sleep quality

According to the method reported in the China Sleep Index Report (Chinese Medical Doctor Association, 2014), we simultaneously consider the sleep status, sleep self-management, and sleep environment to evaluate the sleep quality. The measurement indicators of sleep quality here are based on the Chinese Sleep Index Report. For example, we assess the actual sleep length to judge whether it is easy to wake up at night. The total score of sleep quality was comprehensively calculated. Total score is equal to 100 points, and 64.3 points was the passing score of sleep. The higher the sleep score, the better the sleep quality.

#### Data statistical methods

The neural network model (McCulloch and Pitts, 1943) is a representative machine learning technology, which can automatically learn and output high-level features (Wang, 2018). It is widely used in many research fields (Wang, 2020), such as computer vision, speech recognition, natural language processing, classification prediction; and therefore, it is adopted in this study to study and predict the use time of smartphones before bedtime.

Generally, neural network learning method can be categorized into two types: supervised learning and unsupervised learning (Shengchun and Linxiang, 2006). Among them, in supervised learning, the samples are of known categories. The training samples are input at the input end of the neural network, and then output from the output end through the neural network. By optimizing parameters, the error signal between the actual output and the expected output can be continuously reduced, until it converges to a certain value (Xie, 2015). If the training samples are of unknown categories, the model learning method belongs to unsupervised learning. In this study, since the samples are labeled with five categories of smartphone usage time, the learning method of the neural network is supervised learning.

**Figure 1:**
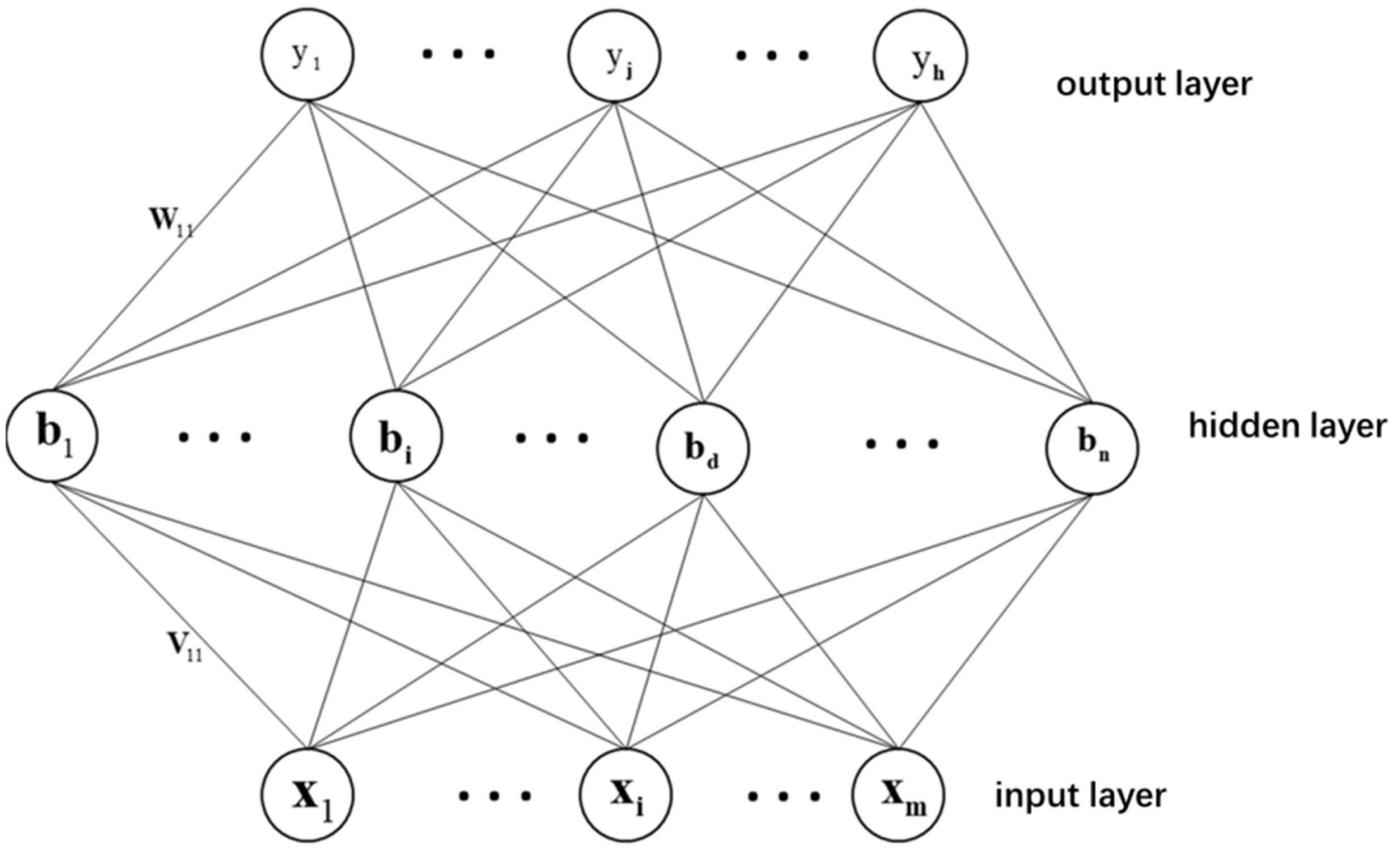
Schematic of the neural network.

The neural network can be divided into three network layers, namely the input layer, hidden layer, and output layer (Rosenblatt, 1958). Each layer contains multiple neurons, and each neuron is linked with the adjacent neurons of previous layer. Neurons can also be divided into three types according to their location. Among them, the neurons in the input layer mainly transmit information from the outside world into the neural network. These neurons do no participate in any calculations, but only transmit necessary information. The neurons located in the hidden layer have an indirect relation with the outside world through the front input layer and back output layer (Hopfield, 1982). They perform calculations, transform the input information of the input layer through the calculation, and then output it to the output layer. The neurons in the output layer output the information from the hidden layer to the outside, that is, to generate the final result (Hinton and Salakhutdinov, 2006). Among them, there is no connection between neurons in the same layer. The schematic diagram is shown in Figure 1.

In Figure 1, $v_{\mathrm{id}}$ refers to the connection weight of the input layer neuron node *i* and the hidden layer neuron node *d*, and $w_{dj}$ represents the connection weight of the hidden layer neuron node d and the output layer neuron node *j*. The input connected to the hidden layer node *d* can then be expressed as


(1) $$a_{d}=\overset{m}{\underset{i=1}{\sum }}v_{\mathrm{id}}\cdot x_{i}$$

And the output received by the output layer node *j* can be written as


(2) $$\beta_{j}=\overset{d}{\underset{h=1}{\sum }}w_{hd}\cdot b_{h}$$

Therefore, for the training set sample $\left(x_{k},\;y_{k}\right)\text{,}$ if the output is equal to ${\hat{y}}_{k}=\;{\hat{(y}}_{1}^{k},{\hat{y}}_{2}^{k},\ldots ,{\hat{y}}_{3}^{k},\;)\text{,}$ we can obtain


(3) $${\hat{y}}_{j}^{k}=f\left(\beta_{j}-\theta_{j}\right)$$

The activation function $f\left(\cdot \right)$ introduces the nonlinearity into the neuron’s output. The hyperbolic tangent function, sigmoid function, and RELU function are three commonly adopted activation functions. The neural network model is a blackbox model (Wang et al., 2019). During the learning and prediction process of the model, the connection weights between neurons are continuously adjusted according to the input training samples.

## Descriptive analysis

### Smartphone usage

**Figure 2:**
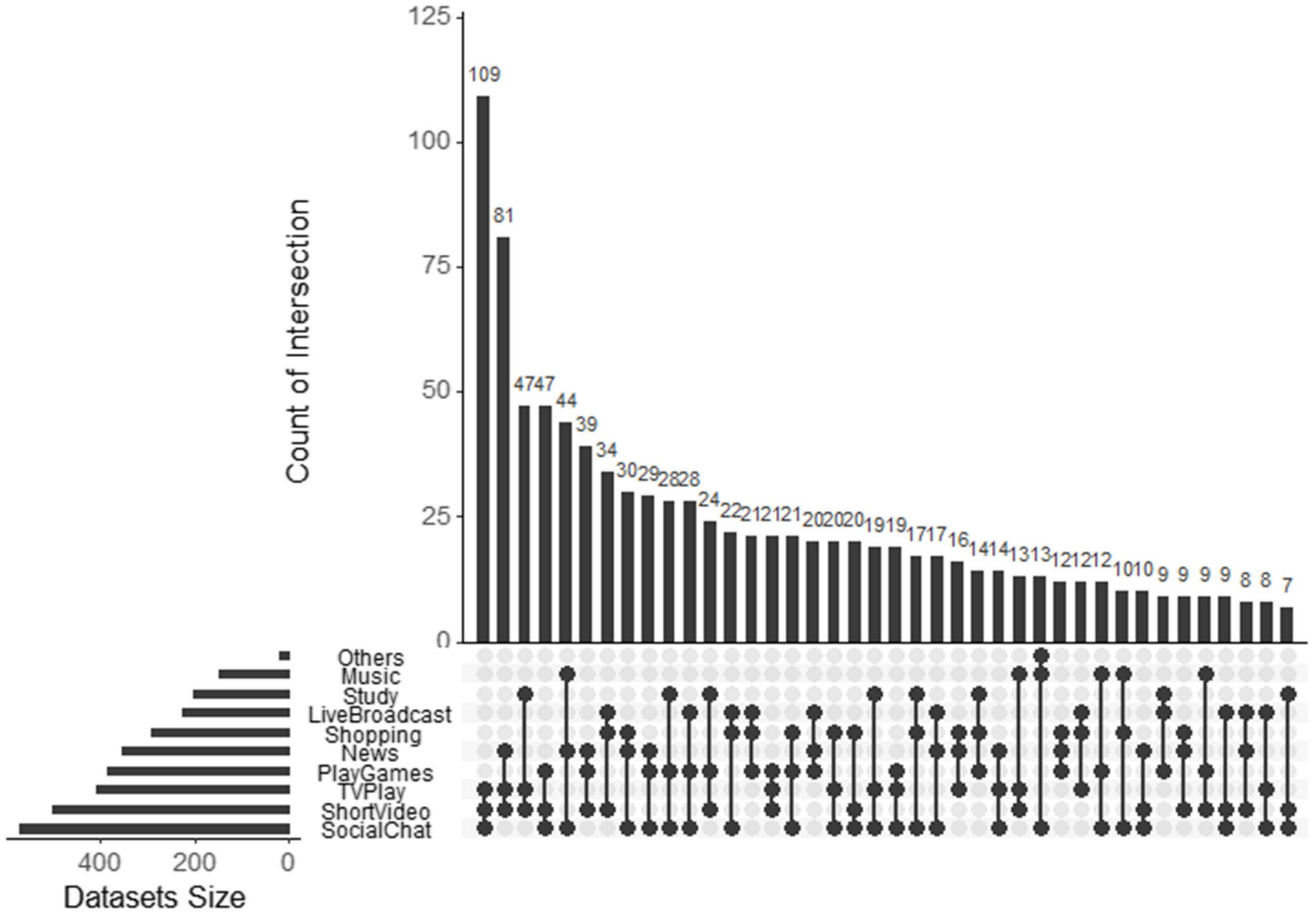
Smartphone use the upset diagram.

According to the survey data, only 5.3% of the participants said that they used smartphones for less than half an hour before going to bed, 24.7% of the students used their smartphones for 0.5 to 1 h, and 49.5% of the students used their smartphones for 2 h and 3 h. It accounted for 13.3 and 7.1% for more than 3 h. The time and frequency of different purposes of using smartphone is then investigated. R software was used to generate an upset plot that includes more than 20 group combination patterns (Conway et al., 2017). The upset shows the relative prevalence of smartphone use for different purposes before bedtime. Among them, social chat is the most common purposes for using smartphone before bedtime (55.02%), followed by short videos (48.26%) and TV shows (39.48%), as shown in Figure 2. Results suggest that social chatting, watching short videos, and watching TV shows are three most frequently used combination patterns in the study, accounting for 10.5% of the 1,035 participants. They are followed by watching short videos, watching news, and watching TV programs, accounting for 8.16%. Next is the combination pattern of studying, watching short videos, and watching news, accounting for 4.54%. The combination of social chatting, watching short videos, and playing games also accounts for 4.54%.

**Figure 3:**
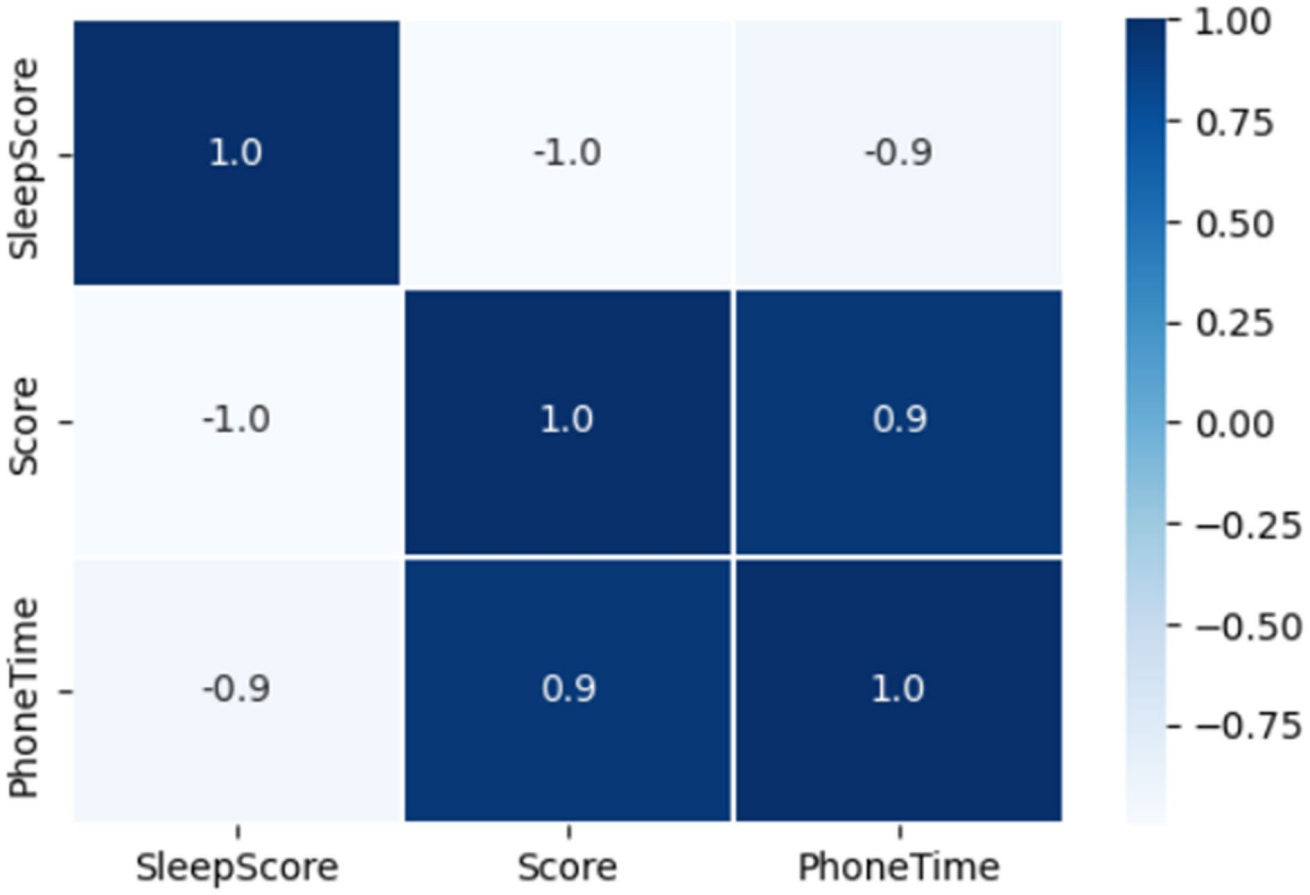
Heatmap.

Large numbers of studies have confirmed that there is a significant correlation between the use of electronic products and sleep problems (Li et al., 2016). Figure 3 shows the heatmap of the smartphone use time, smartphone addiction and sleep quality. It can be seen that there is a close correlation among them. The sleep quality is negatively related to the smartphone use time and smartphone addictive behavior. In other words, the longer the smartphone use before bedtime, the higher the smartphone addiction and the worse the sleep quality. Excessive use of smartphones before going to bed can cause addiction to smartphones, which in turn will have a series of effects on students’ sleep quality. So how to use the smartphone before bedtime is more likely to cause smartphone addiction and smartphone addiction? This will be explored in depth in the Model and Discussion Section.

### The gender difference in using smartphone

A significant test was performed to assess the gender differences in using smartphone (Monacis et al., 2017; Fabris et al., 2020; Longobardi, 2020; Su et al., 2020). According to the ANCOVA results (Table 1), the tested *p* value is less than 0.05, which demonstrates that the use of smartphones of participants are different from those of the female participants. Among them, the female participants are more likely to overuse smartphones because of TV shows and online shopping, while the male participants are more likely to indulge in smartphones due to phone game. This can be attributed to the rapid development of game apps on the market. However, there is no significant difference between the male and female participants in the use of short videos. The use of smartphone to watch short videos have a strong impact on the excessive use of smartphones, regardless of boys and girls.

**Table 1:** Gender ANCOVA results.

Variable	Mean square	F	Significance
Phone usage time	0.300	0.345	0.557
Social chat	0.167	0.672	0.312
Short video	4.084	16.585	0.420
Game	25.077	119.576	0.000
TV show	25.280	117.614	0.000
Music	0.700	5.768	0.016
News	0.927	4.123	0.043
Study	0.398	2.530	0.112
Live streaming	4.995	30.179	0.000
Online shopping	11.363	58.983	0.000
Others	0.115	6.073	0.014

## Model and discussion

### Neural network model

First, neural network model is a typical predictive method in deep learning. It has been introduced in detail in 2.2.3, which includes the input layer, hidden layer and output layer. The data are input through the input layer, and the model will be automatically trained. Finally, the model gives the best predicted result through the output layer. So far, this study has been explored that how to use the smartphone before going to bed has a strong correlation with whether it is addicted to the smartphone before going to bed. In this section, we will study the influence of different uses of smartphones before bedtime on smartphone addiction using the neural network model.

Using this model, the phone use before bed is considered as the input variable, and the output variable is the duration of smartphone use. That is, according to the phone usages before going to bed, the model can be used to make a more accurate prediction of the user’s smartphone usage time. The specific process is described as follows. Small imbalanced datasets are processed using Omission Cross Validation (LOOCV) and Synthetic Minority Oversampling Technique (SMOTE; Chawla et al., 2002) before using a supervised classification algorithm to detect smartphone usage time. Taking the sampling method of SMOTE, a new sample can be synthesized between a given sample and one of its neighboring samples, and in this way, the problem of overfitting can be avoided. Missing cross-validation is an extreme case of k-fold cross-validation, where the model is trained on N-1 data points and tested on one missing sample. Here, N is the total number of samples. There are N iterations in total so that each data point forms the test set once, this method is suitable for small data sets. In this section, the neural network model of multi-classification problem is established where the duration of the use of smartphone is taken as a predictor variable. The five-layer neural network model was initially selected for classification and prediction. The results show that the accuracy rate of the duration of each combination mode is less than 50%, and this model has a poor prediction effect. Then, the ten-layer neural network model was adopted with the activation function. By continuously adjusting the model parameters, the classification accuracy for the first and fifth categories can rise to 99%. The prediction results are listed in Table 2. For the second, third, and fourth categories, although the prediction effect of the ten-layer neural network model is improved significantly as compared to the initial five-layer neural network model, the accuracy rates are still less than 75%.

**Table 2:** Predicted results of neural network model.

Category	Phone use time	Accuracy rate	Recall rate	F1-score
1	<0.5 h	0.98	0.11	0.19
2	0.5–1 h	0.64	0.62	0.63
3	1–2 h	0.69	0.86	0.77
4	2–3 h	0.70	0.85	0.77
5	>3 h	0.99	0.02	0.03

### Model results

We first use the neural network method to predict the duration of smartphone use before bedtime of all categories. The result shows that the prediction accuracy rate is equal to 90%. This illustrates that the neural network model has a high overall accuracy for the duration of smartphone usage. To analyze the usage characteristics among different categories, the neural network method is then used in each category. In this case, the prediction model can be considered as a multi-class prediction problem. As shown in Table 2, different categories have different accuracy rate. To be specific, the highest accuracy rate is as high as 99%, while the lowest accuracy is only about 65%. The high accuracy of the prediction model indicates that the corresponding category exhibits significant features.

**Figure 4:**
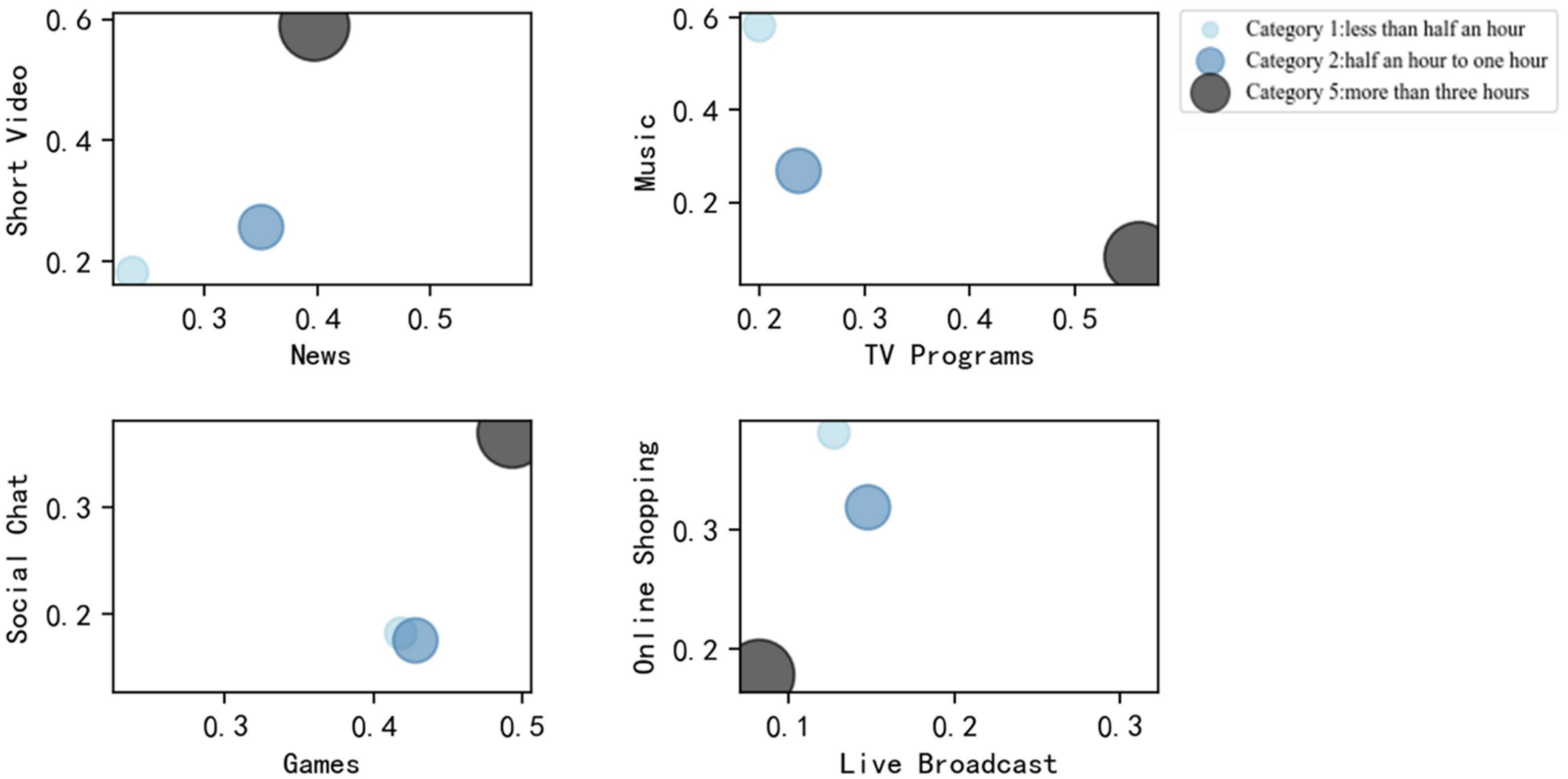
Bubble graph for three typical categories.

Figure 4 plots the bubble graph for three typical categories of smartphone use time, where the eight most frequent smartphone usages are considered including watch short video, watch news, listen to music, watch TV program, social chat, play game, shop online, and watch live broadcasts. The horizontal and longitudinal coordinates represent the time proportion for each smartphone usage. It can be observed that the time proportions for watching short video and listening to music are more than 60% in the first and fifth categories, respectively. The neural network model can work well for these groups with significant characteristics, and therefore, the corresponding prediction accuracy is very high. On the contrary, the second category with the lowest prediction accuracy shows no remarkable characteristic. In this category, the time proportions of eight smartphone usages are all less than 45%. Moreover, the duration of smartphone use in the fifth category is more than 3h. According to the smartphone addiction scale method, it has a very high possibility of addicting to smartphone compared to other categories. As shown in Figure 4, watching short video and playing game take large proportions in the fifth category. This indicates that college students would be lost in smartphone and forget time before bedtime when they watch short video or play game using smartphone. However, for the first category with phone usage time less than 1h, a large proportion of participants tend to listen to music before going to bed. In general, the neural network method is capable of predicting the smartphone usage time, and watching short videos and playing games are more likely to lead to mobile phone addiction behavior before bedtime.

### Discussion

This study investigates the relation of duration of smartphone use before bedtime, sleep quality, and smartphone addiction. The neural network model is implemented to predict the smartphone use behavior.

The results show that the smartphone use time is closely associated with the smartphone behavior, and the resulting smartphone addition behavior has a significant influence on the sleep quality. The phone addition could degrade quality the sleep quality, and even lead to a series of sleep problems. These observations are consistent with previous studies (Qiangqiang, 2021). In practice, for college students, it is necessary to reduce the smartphone usage time to avoid the smartphone addiction behavior.

For the research on smartphone addiction, most of the previous studies focus on the qualitative analysis of smartphone use data (Liu et al., 2017). In this paper, the effect of smartphone use before bedtime on smartphone addiction behaviors among Chinese college students are quantitatively explored. A predictive neural network model is proposed to predict smartphone usage time. Here the duration of smartphone use is discretized (Davide et al., 2022), and the time prediction can then be transformed into a multi-class prediction problem. The neural network model can be adopted to predict the usage time of the mobile phone according to the usage of the mobile phone.

Further, the difference of the model prediction effect in different categories is analyzed. The results indicate that the prediction accuracy of the fifth and first categories are extremely high, which can be attributed to the smartphone usage characteristics. It is found that watching short videos and playing games before going to bed are more likely to cause smartphone addiction behavior, whereas listening to music can help reduce smartphone use time.

In addition, our study also found that there exist significant gender differences in smartphone use before bedtime. Previous studies have demonstrated that female college students have a higher risk of addicting to smartphone as compared to male college students (Roberts et al., 2015; Lingling and Zhiqiang, 2017). This study found that men and women also differ in the smartphone usage before bedtime. Female participants are more likely to be addicted to smartphone because of watching TV programs, while male participants are more likely to use smartphone to play games.

## Suggestions

In recent years, with the popularization of smartphones, the smartphone addiction phenomenon has gradually increased (Cain et al., 2011). For college students, excessive use of smartphones has significantly affected their daily study (Li et al., 2015). College students in the mobile world have a relationship between people and people (Wang et al., 2015). It becomes a symbolic virtual communication, which can also cause cognitive distortion (Wu, 2018). It is of great importance to guide college students to use smartphones correctly and avoid smartphone addiction. Based on the results in this work, the following recommendations are put forward:

For college student, excessive use of smartphones before bed can cause the smartphone addiction behavior. It is necessary to guide students to use smartphones correctly and avoid the negative impact of smartphones. As to school, the schools can offer quality training courses on smartphone networks and other aspects according to the actual situation and help guide the students to correctly use the smartphone. From the perspective of college students, students should learn to plan their time reasonably. As to the smartphone user, make a long-term plan to rationally use time, which can effectively prevent aimless loss of direction and avoid addiction to smartphones. At the same time, actively participating in school activities and integrating into group activities will help reduce dependence on smartphones.

Moreover, the features such as smartphone recommendations, have a huge impact on students’ smartphone addiction. Especially with the rise of short videos and the improvement of smartphone software push functions, users are constantly pushing interesting content to induce students to continue to indulge in them. In schools, a series of guidance courses are carried out to make students understand the dangers of excessive use of smartphone games and short videos, correctly guide college students to use smartphones rationally, and reduce the use of games and short videos. In terms of students themselves, students should also continuously strengthen their restraint ability and use smartphones reasonably and correctly.

There are some limitations in this study. The subjects of this study are college students. In fact, apart from college students, other groups with different occupations and ages can be studied and compared in the future. Moreover, the data in this study are horizontal data, and longitudinal data can be considered in the future research.

## Data availability statement

The original contributions presented in the study are included in the article/supplementary material; further inquiries can be directed to the corresponding author.

## Ethics statement

Written informed consent was obtained from the individual(s) for the publication of any potentially identifiable images or data included in this article.

## Author contributions

LW contributed to conceptualizing ideas, implementing the studies, and writing the initial draft. LL contributed to mentoring, acquiring funding, and manuscript revision. XW contributed to acquiring funding and manuscript revision. All authors contributed to the article and approved the submitted version.

## Funding

This work was supported by the Social Science Planning Project of Shandong Province, China (grant no. 21CTQJ03).

## Conflict of interest

The authors declare that the research was conducted in the absence of any commercial or financial relationships that could be construed as a potential conflict of interest.

## Publisher’s note

All claims expressed in this article are solely those of the authors and do not necessarily represent those of their affiliated organizations, or those of the publisher, the editors and the reviewers. Any product that may be evaluated in this article, or claim that may be made by its manufacturer, is not guaranteed or endorsed by the publisher.
